# M2e/NP Dual Epitope-Displaying Nanoparticles Enhance Cross-Protection of Recombinant HA Influenza Vaccine: A Universal Boosting Strategy

**DOI:** 10.3390/vaccines13040412

**Published:** 2025-04-15

**Authors:** Rui Liu, Lejun Yang, Jin Feng, Songchen Zhang, Liping Wu, Yingying Du, Dexin Kong, Yuhua Xu, Tao Peng

**Affiliations:** 1 State Key Laboratory of Respiratory Diseases, Sino-French Hoffmann Institute, School of Basic Medical Sciences, Guangzhou Medical University, Guangzhou 511436, China; liurui1@ycszxrmyy.wecom.work (R.L.); 2022210174@stu.gzhmu.edu.cn (L.Y.); fengjin@wmmqy1264680.wecom.work (J.F.); 2022210033@stu.gzhmu.edu.cn (S.Z.);; 2 Guangdong South China Vaccine Co., Ltd., Guangzhou 510663, China; wuliping@gdscvc.com (L.W.); xuyuhua@gdscvc.com (Y.X.)

**Keywords:** universal influenza vaccine, enhanced recombinant HA influenza vaccine, nucleoprotein (NP), matrix 2 ectodomain (M2e), M2e/NP epitope display, broad protection, adjuvant, nanoparticle

## Abstract

**Background/Objectives**: Vaccination remains the most effective means of preventing influenza virus infections. However, the continuous antigenic drift and shift of influenza viruses lead to a reduced efficacy of the existing vaccines, necessitating vaccines capable of broad protection. **Methods**: To address this, we developed a modular vaccine strategy pairing a clinical-stage adjuvanted recombinant hemagglutinin (HA) vaccine (SCVC101) with OMN, a heptameric nanoparticle displaying conserved influenza A virus T-cell epitopes from nucleoprotein (NP) and matrix 2 ectodomain (M2e). **Results**: OMN induced cross-reactive M2e-specific antibodies, binding to diverse influenza A subtypes. Critically, the co-administration of OMN with SCVC101 enhanced cellular immunity and cross-protection without diminishing HA-induced humoral responses. **Conclusions**: This dual-antigen delivery system enables annual HA component updates, aligned with WHO recommendations, while the conserved OMN nanoparticle acts as a universal booster, leveraging existing production infrastructure. This approach offers a promising strategy for improving the influenza vaccine’s efficacy against emerging viral variants.

## 1. Introduction

Despite the availability of multiple influenza vaccines and therapeutic antivirals, seasonal influenza viruses continue to infect 5~15% of the global population each year, resulting in hundreds of thousands of deaths [1]. As RNA viruses, influenza viruses have segmented genomes and error-prone RNA-dependent RNA polymerases, which allow for frequent antigenic drifts and, occasionally, antigenic shifts in influenza viruses [2], enabling them to evade the immune system. This, coupled with the lag in the development and production of influenza vaccines, has resulted in a situation where existing vaccines are often not optimally effective in preventing influenza infections, particularly when there is an antigenic mismatch between the vaccine strains and the circulating strains [3].

Conserved proteins or fragments of influenza A viruses, such as the nucleoprotein (NP) and the extracellular domain of matrix protein 2 (M2e) [4,5,6,7], are regarded as effective targets for the design of universal influenza vaccines. As a transmembrane protein, M2 functions as a pH-regulated ion channel critical for mediating proton influx during viral uncoating, thereby enabling efficient genomic release into host cells during influenza infections [8]. Comprising 23 amino acids, M2e is highly conserved among influenza A virus strains and can induce a broad-spectrum protection against heterosubtypic influenza viruses. However, in its natural state, the immunogenicity and abundance of M2e are low [9]. A multivalent display of M2e epitopes through tandem repeats coupled with immunogenic carriers significantly augments its antigenicity, as demonstrated by enhanced antibody titers and T-cell activation profiles [10,11,12]. Conversely, NP, a structurally conserved influenza core component, undergoes proteasomal processing in infected host cells. Processed NP-derived epitopes are subsequently loaded onto MHC class I molecules for surface presentation. This NP-MHC I complex engages cognate TCRs on circulating CD8+ T lymphocytes, triggering clonal expansion and differentiation into cytotoxic effectors. These activated CTLs execute viral clearance through perforin–granzyme-mediated cytolysis and Fas/FasL-dependent apoptosis pathways, establishing robust cell-mediated immunity against influenza infection [13,14]. The presence of conserved epitopes in the influenza virus NP allows for the cross-reactivity of CTLs, enabling them to target different influenza subtypes, which is a critical factor in controlling influenza virus infections. This cross-reactivity aids in inhibiting virion release, controlling viral replication, and reducing the severity of influenza-related diseases [15].

Self-assembly nanotechnology represents a promising strategy that may facilitate the creation of cross-protective influenza vaccines by displaying cross-protective epitopes on nanoparticles and enhancing their immunogenicity. Substantial evidence indicates that antigens formulated as nanoparticles demonstrate greater efficacy than their monomeric soluble counterparts [16]. Nanoparticles enhance the uptake of the anchored antigen by antigen-presenting cells [17]. Numerous natural protein nanoparticles have exhibited capabilities for antigen presentation and immune stimulation [18,19]. OVX313, a protein domain comprising 55 amino acids, can self-assemble into heptameric nanoparticles [20]. The nanoparticle vaccine OVX836, utilizing OVX313 as a carrier, can induce the production of a substantial number of NP-specific lung CD8+ tissue-resident memory T cells, which confer long-term resistance to influenza viruses [21,22]. Furthermore, clinical studies have demonstrated that it elicits both humoral and cellular immune responses [23].

In this study, we screened peptides that represent immunodominant epitopes for CTL and Th responses within the NP protein using immunoinformatic methods. These peptides were then linked to multiple tandem M2e and fused with the OVX313 heptamer to create a carrier that self-assembled into heptameric nanoparticles (OMNs). Mice immunized with OMN produced elevated levels of M2e-specific antibodies, which demonstrated varying degrees of binding to M2e fragments across five subtypes of the influenza A virus. Additionally, we supplemented the OMN vaccine with the previously reported recombinant tetravalent hemagglutinin influenza vaccine SCVC101 [24] that we have developed to assess their effects on both humoral and cellular anti-influenza virus immunity in mice. The results indicate that OMN enhanced the cellular immunity and cross-protection capabilities of SCVC101 without compromising its humoral immunity.

## 2. Materials and Methods

### 2.1. Viruses, Competent Cells, and Animals

A/Victoria/2570/2019 (H1N1) was provided by Guangdong South China Vaccine Co., Ltd. (Guangzhou, China), while A/PuertoRico/8/34 (H1N1) was obtained from Foshan Huamiao Pharmaceutical Technology Co., Ltd. (Foshan, China). The BL21(DE3) and DH5α competent cells were purchased from Takara (Kusatsu, Japan). Female BALB/c mice, aged 7 to 9 weeks, were acquired from Guangdong Vitong Lihua Experimental Animal Technology Co., Ltd. (Foshan, China). All animal surgeries were approved by the Experimental Animal Use and Management Committee of Foshan Huamiao Pharmaceutical Technology Co., Ltd. (Foshan, China), and were conducted in accordance with its guidelines.

### 2.2. NP Protein T-Cell Epitope Prediction

Using NetCTL1.2 (http://www.cbs.dtu.dk/services/NetCTL/ (last accessed on 15 December 2022)), IEDB-MHCI (http://tools.immuneepitope.org/mhci/ (last accessed on 15 December 2022)), and SYFPEITHI (http://www.syfpeithi.de/ (last accessed on 15 December 2022)), we predicted CTL epitopes in the NP protein of the A/Darwin/6/2021 (H3N2) strain. To perform this analysis, we entered the full-length amino acid sequence of NP, selected ’human’ as the species, and chose the dominant allele HLA-A*02:01 [25], which represents the global population. We set the peptide length to 9 and identified the top 14 epitopes as candidate epitopes. Additionally, for the prediction of Th epitopes in the NP protein of A/Darwin/6/2021 (H3N2), we utilized IEDB-MHCII (http://tools.immuneepitope.org/mhcii/ (last accessed on 15 December 2022)) and RANKPEP (http://imed.med.ucm.es/Tools/rankpep.html (last accessed on 15 December 2022)). In this case, we again entered the full-length amino acid sequence of NP and selected the dominant allele HLA-DRB1*0101 [25], which is representative of the world population. The default parameters were applied to the remaining options, resulting in the selection of the top 15 epitopes as candidates. Furthermore, based on the predicted distribution of the target peptide across the entire NP sequence, a frequency distribution histogram was generated using GraphPad Prism 9.0.

### 2.3. Construction of OMN Protein Expression Vector

The recombinant protein OMN consists of OVX313 at the N-terminal, followed by six tandem M2e fragments (SLLTEVETPIRNEWGSRSNDSSD, derived from A/Cambodia/e0826360/2020 (H3N2)), an NP2 fragment (amino acids 245-400, derived from A/Darwin/6/2021 (H3N2)), and the OVX313 heptamerization domain (KKQGDADVCGEVAYIQSVVSDCHVPTAELRTLLEIRKLFLEIQKLKVEGRRRRRS) [19]. The cysteine residues at positions 16 and 18 of M2e were changed to serine to prevent the formation of undesirable disulfide bonds between the peptide chains [26]. Each component is linked by a flexible linker (GGGSG/G4S) to minimize steric hindrance. Additionally, to facilitate subsequent purification, an 8×His tag was appended to the C-terminus of the OMN protein sequence. The optimized sequence was cloned into the pET-28(+) vector and synthesized by GenScript for recombinant expression.

### 2.4. OMN Protein Expression and Purification

Following the transformation of the recombinant plasmid into the *E. coli* BL21(DE3) competent strain, transformants were cultured in LB medium supplemented with antibiotics. Protein expression was induced by adding 0.5 mM IPTG when the culture reached an OD600 of 0.6. Bacterial cells were harvested by centrifugation (8000× *g*, 10 min, 4 °C) and lysed via probe sonication in an ice-water bath under optimized parameters (300 W power, 5 s on/10 s off pulse cycles for 15 min). As OMN predominantly formed insoluble inclusion bodies, the particulate fraction was isolated through differential centrifugation (12,000× *g*, 20 min). The pellet was subjected to one cycle of resuspension in denaturing wash buffer (2 M urea, 50 mM Tris-HCl, 500 mM NaCl, 0.1% Triton X-100, pH 8.5), followed by centrifugation to remove the soluble contaminants. The washed inclusion bodies were subsequently resuspended in a denaturing buffer (8 M urea, 50 mM Tris, 500 mM NaCl, pH 8.5). Ni2+ column (Chelating Sepharose Fast Flow, GE Healthcare, Chicago, IL, USA) was applied for affinity chromatography, followed by washing with a denaturing elution buffer (8 M urea, 50 mM Tris, 500 mM NaCl, 300 mM imidazole, pH 8.5) to elute the bound protein. Finally, a desalting column (HiTrap Desalting, GE Healthcare, USA) was utilized to remove the imidazole and urea, facilitating fluid replacement and refolding.

### 2.5. SDS-PAGE, Western Blot, and Dynamic Light Scattering

For SDS-PAGE, the untreated purified OMN were analyzed under reducing conditions with 4–12% polyacrylamide gels (GenScript Biotech Co., Ltd., Piscataway, NJ, USA). Subsequently, a gel analysis system was employed to calculate the gray value of the target protein and assess its purity.

For Western blotting, OMN proteins were transferred to nitrocellulose membranes via semi-dry transfer. The membranes were blocked with 10% skim milk/PBS (pH 7.4) for 1 h, then incubated with mouse anti-His mAb (1:1000) at 25 °C. After the PBST washes, the blots were probed with an AP-conjugated goat anti-mouse IgG (1:10,000) and detected using Tanon™ ECL substrate following the manufacturer’s protocol (signal acquired within 5 min).

For dynamic light scattering (DLS), the OMN nanoparticle size distribution was analyzed by DLS using a NanoBrook Omni system ( Brookhaven Instruments, Nashua, NH, USA). The filtered samples (0.22 μm) in quartz cuvettes (10 mm path length) were measured in triplicate at 25 °C (a 90° scattering angle). The hydrodynamic radii were calculated via the Stokes–Einstein equation using intensity-weighted data.

### 2.6. Immunization and Viral Infection Challenge in Mice

To evaluate the OMN-induced cross-reactive antibody production in mice, female BALB/c mice (*n* = 6 per group) were immunized intramuscularly with OMN (10 μg/mouse) or PBS on days 0, 21, and 42. Blood samples were collected for testing on day 62. In the co-administration of OMN and the seasonal influenza vaccine SCVC101 experiment, the female BALB/c mice are immunized twice with OMN (10 μg/mouse), or SCVC101 (1.5 μg/HA), or co-administered OMN (10 μg/mouse) and SCVC101 (1.5 μg/HA), at an interval of 21 days. PBS serves as the control group (n = 25 per group). The SCVC101 vaccine was formulated with recombinant HA (1.5 μg rHA/each strain of virus/dose, 25 μL/dose) based on the WHO-recommended HA gene sequences for the Northern Hemisphere 2021–2022 influenza season and adjuvanted with CD-A (25 μL/dose) [24], a MF59-like oil-in-water emulsion containing squalene. On the 20th day after the last immunization, splenocytes were harvested for an ELISpot analysis. On day 42, half of the mice were intranasally infected with a lethal dose of the A/Victoria/2570/2019 (H1N1) virus (1 × 10^6^ TCID50/mouse), while the other half were intranasally infected with a lethal dose of the H1N1 (PR8) virus (5.62 × 10^5^ TCID50/mouse). Six days post-challenge, the right lungs were harvested for a virus titration, and changes in the body weight and survival rates of the mice were monitored for two weeks following the challenge. Mice that experienced a weight loss exceeding 25% during the infection were euthanized.

### 2.7. ELISA

The levels of IgG, IgG1, or IgG2a were measured in the serum samples collected on day 41. The 96-well ELISA plates were pre-coated with 100 ng of one of the following proteins or peptides: OMN, M2e (A/Puerto Rico/30/2022, A/Cambodia/e0826360/2020, A/Guangdong-Shenzhen/1/2011, A/Jiangsu/602/2021, A/Jiangxi-Donghu/346-2/2013), NP2-1 (AEIEDLIFLARSALILRGSVAHKS), NP2-2 (SLVGIDPFKLLQNSQIYSLIRPNEN), NP2-3 (RLLSFIRGTKVSPRGKLSTRGVQIA), NP2-4 (NMGSSTLELRSGYWAIRTRSGGNTN), or rHA (H1N1 A/Victoria/2570/2019, H3N2 A/Cambodia/E0826360/2020, B/Victoria/Washington/02/2019, or B/Yamagata/Phuket/3073/2013) overnight at 4 °C. The serum samples from the immunized mice were subjected to serial two-fold dilutions (100 μL/well) and incubated at 37 °C for 1 h. Antigen-bound antibodies were detected using an HRP-conjugated goat anti-mouse IgG (1:10,000; Abcam, Cambridge, UK), followed by a colorimetric development with 100 μL of tetramethylbenzidine (TMB) substrate per well. The absorbance at 450 nm was measured using an ELISA plate reader (Botten Instrument Co., Ltd., Kunshan, China). For the mouse serum cross-antibody reaction, the maximum dilution factor of 1:213 was used. For the co-administration of OMN and SCVC101, the endpoint titers were calculated as the reciprocal of the highest serum dilution that exhibited OD450 values more than two-fold the control value.

### 2.8. ELISpot

To evaluate the specific T-cell responses, the Mouse IFN-γ and IL-4 ELISpotPLUS kit (MabTech, Nacka Strand, Sweden) was used, according to the manufacturer’s instructions. The spots were counted using a CTL Immunospot Reader (Cellular Technology Ltd., Shaker Heights, OH, USA). Spleen isolation and lymphocyte preparation were conducted under sterile conditions. The mouse spleens were homogenized in RPMI 1640 medium using syringe plungers in a 35 mm culture dish. The cell suspension was layered over 4 mL of murine lymphocyte separation medium in a 15 mL conical tube and centrifuged at 400× *g* for 35 min (room temperature, 3-step acceleration/deceleration). The lymphocyte layer was transferred to a new tube, washed twice with RPMI 1640 (300× *g*, 10 min), and resuspended in 1 mL of serum-free medium containing L-glutamine. The cell viability was assessed by mixing 10 μL of cell suspension with 0.08% Trypan blue, followed by automated counting. A total of 1 μg of M2e peptide, NP2-1 peptide, or rHA protein was pre-coated on the ELISpot plate. Subsequently, 2.5 × 10^5^ spleen lymphocytes were added to each well and incubated in a 37 °C, 5% CO_2_ incubator for 36 h. After rinsing with PBS, the wells were treated with PBS containing 0.5% fetal bovine serum (FBS) and incubated at room temperature for 2 h. Following another rinse with PBS, 100 μL of streptavidin-ALP (diluted to 1:1000 in PBS with 0.5% FBS) was added and incubated at room temperature for 1 h. After a subsequent wash with PBS, 100 μL of the substrate solution (BCIP/NBT-plus) was added and kept in the dark at room temperature for 15 min. An ELISpot analyzer was used to count the spots.

### 2.9. Hemagglutinin Inhibition (HAI) Assay

The serum from the immunized mice was treated with a receptor-destroying enzyme (RDE, Nippon Sanken, Tokyo, Japan). The serum was then diluted with PBS, and 25 μL of a calibrated virus solution containing four units was added. The mixture was allowed to stand at room temperature for 30 min. Subsequently, 25 μL of a 1% chicken red blood cell suspension was added to each well, mixed gently by pipetting, and allowed to stand at room temperature for an additional 30 min before observing the results. The inhibitory potency of the red blood cells is defined as the reciprocal of the highest dilution at which hemagglutination is completely inhibited.

### 2.10. Statistical Analysis

Experimental data are presented as a mean ± standard error of the mean (SEM). The statistical analysis and graphing were performed using GraphPad Prism 9.0. The data were analyzed using independent sample *t*-tests or a one-way ANOVA to evaluate the significance of differences between the treatment group and the control group (ns: no significance; *: *p* < 0.05; **: *p* < 0.01; ***: *p* < 0.001; ****: *p* < 0.0001).

## 3. Results

### 3.1. Selection of T-Cell Epitopes from NP Proteins

**Figure 1:**
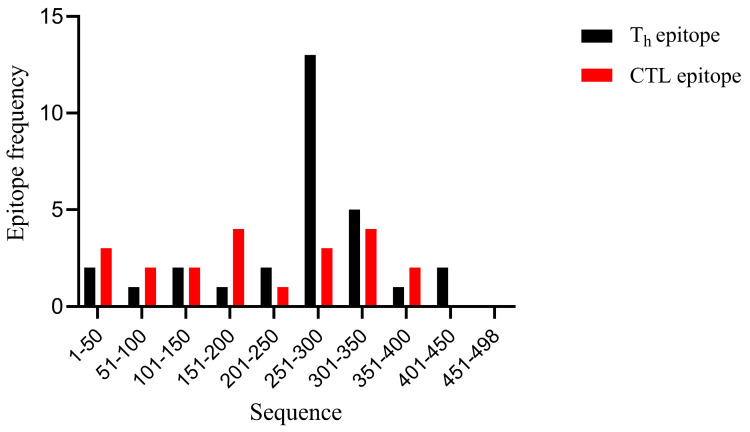
Distribution of T-cell epitopes predicted in the full-length influenza A NP amino acid sequences.

In this study, we utilized four immunoinformatics platforms to identify predicted immune-protective human epitopes. Collectively, these platforms (NetCTL-1.2, IEDB-MHCI, and SYFPEITHI) predicted eight top-ranked CTL epitopes, which are highlighted in Table 1. Additionally, the IEDB-MHC II and RANKPEP websites were employed to predict Th epitopes, resulting in the selection of the top 15 peptides (Table 2). As illustrated in Figure 1, all predicted CTL and Th epitopes within the NP protein were identified, primarily concentrated in the C-terminal half, consistent with previous findings [27]. Notably, the peptides NP366-374 and NP383-391 have been predicted in earlier studies [27,28]. Based on these results, peptide NP245-400 (designated NP2) was chosen as a conserved NP T-cell epitope.

### 3.2. Expression and Characterization of OMN Nanoparticles

**Figure 2:**
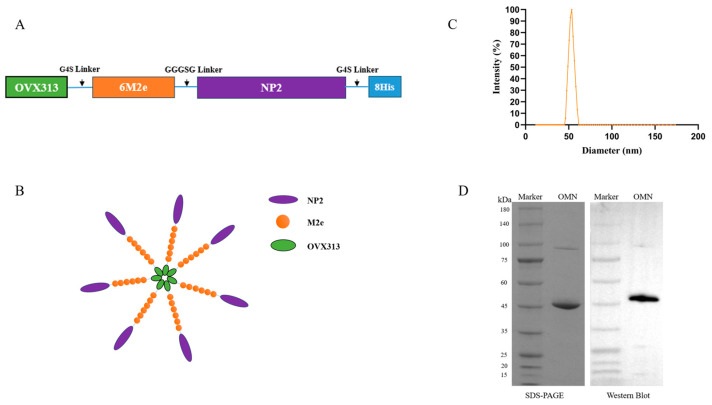
Construction and characterization of OMN nanoparticles. (**A**) OMN: the OVX313 peptide is connected to the N-terminus of six series-connected M2e units, along with one NP2. A His tag is added at the C-terminus, and all components are linked through a flexible linker (GGGSG/G4S) to minimize the steric hindrance. (**B**) Schematic representation of OMN heptamer nanoparticles. (**C**) Dynamic light scattering was employed to determine the average particle size of the OMN. (**D**) SDS-PAGE and Western blot identification of OMN protein.

Six tandem M2e fragments (SLLTEVETPIRNEWGSRSNDSSD, derived from A/Cambodia/e0826360/2020 (H3N2) and one NP2 fragment (245-400 aa, from A/Darwin/6/2021 (H3N2)) were connected to the C terminus of OVX313 (Figure 2A). To prevent the formation of undesired disulfide bonds between peptide chains, the cysteine residues at positions 16 and 18 of M2e were replaced with serine [26]. The components were linked via a GGGSG/G4S flexible linker to minimize the steric hindrance. Figure 2B schematically represents the OMN nanoparticles. Dynamic light scattering (DLS) measures the average particle size at 43 nm (Figure 2C), which is consistent with the previous report [19]. The SDS-PAGE and Western blot analyses (Figure 2D) indicated a purity of about 90% (Figure 2D).

### 3.3. OMN Nanoparticle-Induced Broad Spectrum M2e Antibodies

To be able to analyze antigen-specific T-cell responses, peptides for the M2e (Table 3) and NP (Table 4) proteins were generated. The NP peptides comprise four segments (NP2-1: NP 251-274, NP2-2: NP 297-321, NP2-3: NP 342-366, and NP2-4: NP 373-397) that cover the NP2 region, which was predicted to contain concentrated T-cell epitopes. As shown in Table 3 and Table 4, we compared the M2e and NP amino acid sequences of different influenza A viruses (H1N1, H3N2, H5N1, H9N2, and H10N8) and aligned their sequences with our antigen sequences, with the diversified sequences underlined, while the conserved sequences were bolded. We found that the first eight amino acids of M2e as well as the NP2-1 sequence region are completely conserved among all influenza A subtypes.

**Figure 3:**
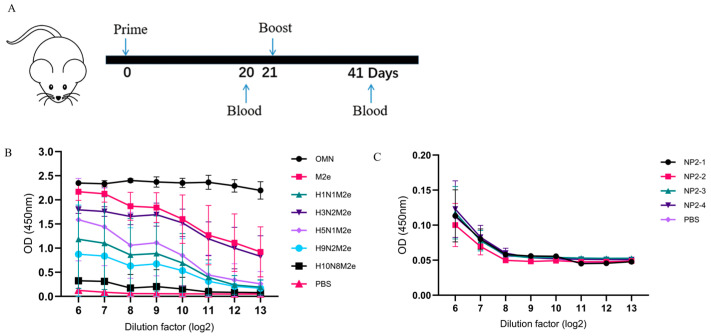
Comparison of breadth of serum antibody responses in immunized mice. (**A**) Immunization and sampling plan. (**B**) The cross-binding ability of the serum following secondary immunization with OMN to the M2e peptide of each subtype of influenza A virus. (**C**) The binding ability of antibodies from NP2 peptides in sera after secondary immunization with OMN. Specifically, immunoinformatics was utilized to predict the T-cell epitopes in the NP protein of the A/Darwin/6/2021 (H3N2) strain. The generated peptide NP2 was truncated into four segments, resulting in the corresponding peptide library (NP2-1, NP2-2, NP2-3, and NP2-4). The ELISA method was selected to detect the IgG antibody levels, with an initial dilution factor of 1:26 and a maximum dilution factor set to 1:213. Absorbance was measured at a wavelength of 450 nm. N = 6.

To assess the immunogenicity of OMN, mice were immunized intramuscularly with OMN (10 μg/mouse) or PBS as a control on day 0, with one boost immunization on day 21 (Figure 3A). Sera collected on day 41 were used to measure specific IgG levels. As shown in Figure 3B, OMN induced the highest level of M2e-specific IgG antibodies against itself. Furthermore, OMN also induced antibodies that recognize the M2e peptides from the other influenza virus A sequences, and the antibody titer correlated with the level of sequence similarities. This suggests that OMN has the potential to induce cross-subtype M2e antibodies, and lower antibody levels were observed when the peptide sequences of the vaccine antigen exhibited reduced homology to the proteins used in ELISA.

The NP-coated peptide groups showed comparable serum IgG levels to the PBS group, indicating a low-level NP-specific IgG production (Figure 3C).

### 3.4. Co-Immunization of SCVC101 and OMN-Induced Strong Cellular Immune Responses

**Figure 4:**
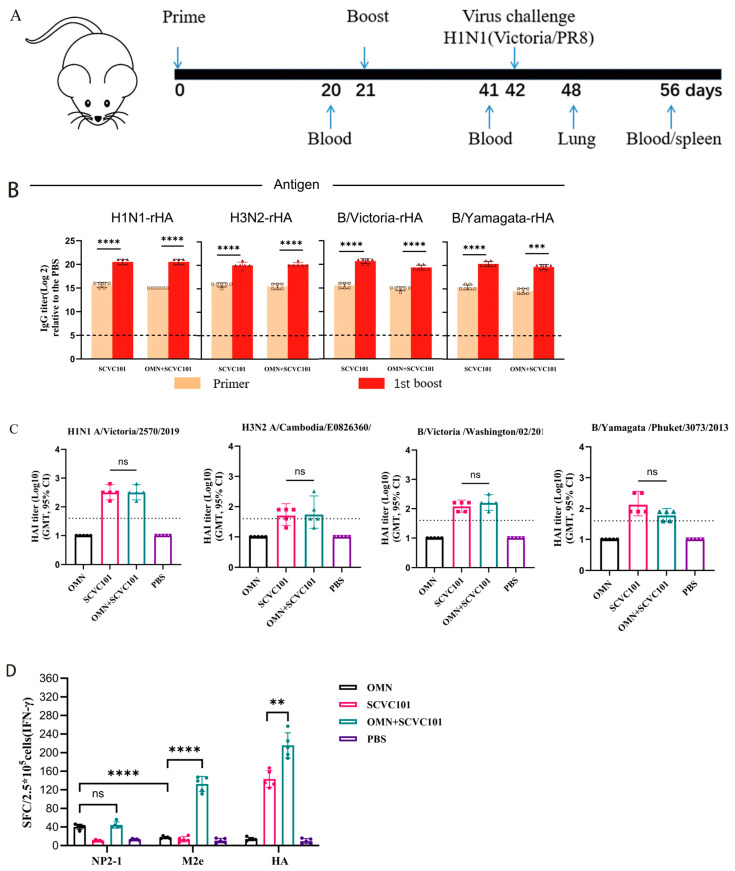

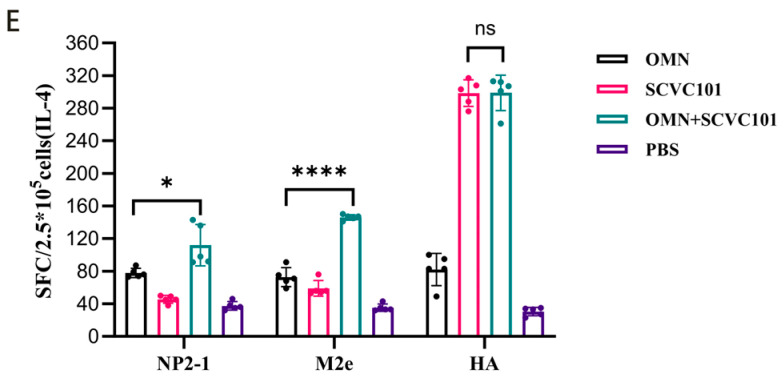
Evaluation of humoral and cellular immunity in mice immunized with SCVC101 and OMN alone or in combination. (**A**) The immunization, sampling, and challenge protocol for the mice is outlined. (**B**) Specific IgG titers in serum were measured three weeks post-immunization, with a sample size of n = 6. (**C**) Hemagglutination inhibition levels of mouse serum against various subtypes of the influenza virus were determined following the final immunization, n = 5. The viruses included the following: H1N1 A/Victoria/2570/2019, H3N2 A/Cambodia/E0826360/2020, B/Victoria/Washington/02/2019, and B/Yamagata/Phuket/3073/2013. The dashed line indicates the effective titer limit for HAI (≥1:40). (**D**,**E**) The ELISpot method was employed to quantify the levels of IFN-γ and IL-4 secreted by spleen lymphocytes, with a sample size of n = 5, including (**D**) the number of splenocytes secreting IFN-γ and (**E**) the number of splenocytes secreting IL-4. (ns: no significance; *: *p* < 0.05; **: *p* < 0.01; ***: *p* < 0.001; ****: *p* < 0.0001).

SCVC101 is a clinical-stage quadrivalent rHA-based influenza vaccine [24]. We sought to determine whether supplementing SCVC101 with OMN could enhance both the magnitude and breadth of protective immune responses. To achieve this, we first investigated whether OMN would interfere with the immunogenicity of SCVC101. Mice were immunized with (SCVC101+OMN) according to the schedule shown in Figure 4A. Serum samples were collected from the immunized mice on days 20 and 41, and the antibody levels against the four rHAs in SCVC101 were measured using ELISA. As shown in Figure 4B, the addition of OMN did not impair the antibody immunogenicity of SCVC101 against any of the four rHAs.

To further assess the impact of (SCVC101+OMN) immunization on HAI, we conducted an HAI assay against four influenza viruses corresponding to the sequences of the four rHAs: H1N1 A/Victoria/2570/2019, H3N2 A/Cambodia/E0826360/2020, B/Victoria/Washington/02/2019, and B/Yamagata/Phuket/3073/2013. As anticipated, the OMN-only group exhibited no hemagglutination inhibition (Figure 4C). The (SCVC101+OMN) group demonstrated similar levels of HAI activity compared to the SCVC101 group. We next evaluated the impact of the SCVC101 vaccine on T-cell immunogenicity induced by OMN. The splenocyte IFN-γ production was assessed by the ELISpot assay, following stimulation with the NP2-1 peptide, M2e peptide, or rHA protein. As shown in Figure 4D, since SCVC101 does not contain NP2 and M2e sequences, NP2-1- and M2e-specific cellular responses cannot be detected in SCVC101-immunized mice. On the other hand, the combination of SCVC101 and OMN significantly enhanced the IFN-γ responses to M2e peptides and HA proteins, although no significant enhancement was observed for NP2-1. Furthermore, upon stimulation with NP2-1 and M2e peptides (Figure 4E), co-immunization (SCVC101+OMN) augmented IL-4 secretion. These findings demonstrate that the co-administration of SCVC101 and OMN elicits a more robust cellular immune response compared to either vaccine alone.

In summary, OMN did not affect the HA-specific humoral immune response induced by SCVC101. Conversely, SCVC101 notably amplified the T-cell responses triggered by OMN, likely because of the CD-A adjuvant present in SCVC101.

### 3.5. (SCVC101+OMN) Immunization Enhanced Protection Against H1N1 Influenza Virus Infection in Mice

To evaluate the cross-protective effect of combined immunization, we infected mice with lethal doses of either H1N1/Victoria or H1N1/PR8. On the sixth day post-infection, four mice from each group were selected for lung virus titer detection, while the body weight changes and survival rates were monitored daily for two weeks. The mice that lost more than 25% of their body weight were humanely euthanized.

**Figure 5:**
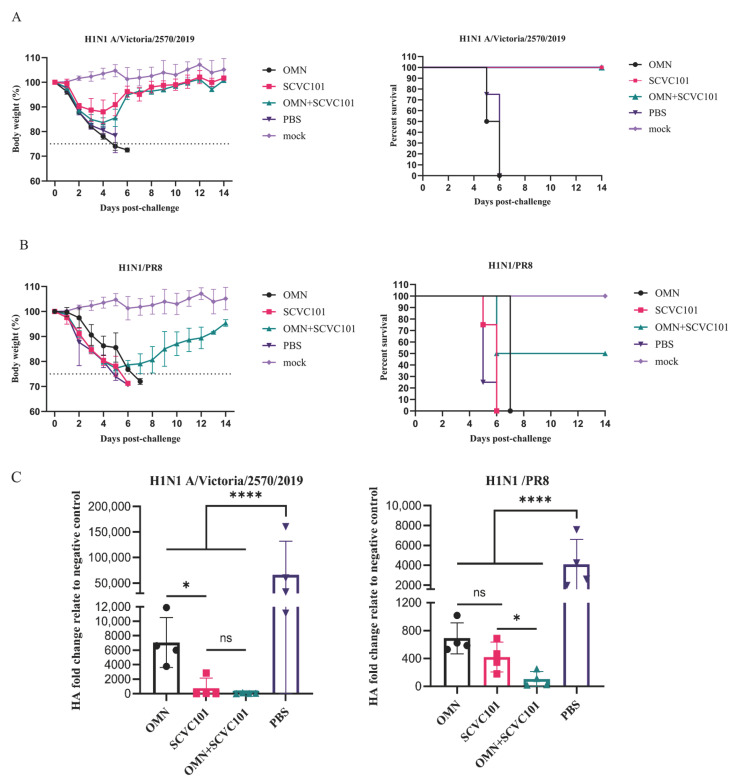
Evaluation of the protection against influenza A virus infection challenge in mice. Twenty-one days following the last immunization, eight mice in each group were intranasally infected with lethal doses of the H1N1/Victoria influenza virus and the H1N1/PR8 influenza virus. Changes in mouse body weight and survival rates during the two weeks following infection with H1N1/Victoria (**A**) and H1N1/PR8 (**B**). (**C**) The qPCR relative quantification method was employed to detect virus titers in the lungs of mice on the sixth day post-infection. (ns: not significant; *: *p* < 0.05; ****: *p* < 0.0001).

In the H1N1/Victoria challenge-infection groups, all PBS-treated mice died within six days, whereas the OMN group exhibited progressive weight loss that necessitated euthanasia by day seven as the body weight dropped below 25%. In contrast, both the SCVC101 mono-immunization and SCVC101+OMN combination groups demonstrated weight recovery starting on day five post-infection, with all mice surviving (Figure 5A). These groups also maintained significantly lower lung virus titers compared to the controls (Figure 5C). For the H1N1/PR8 challenge-infection groups, the PBS-treated mice died within one week, while the OMN and SCVC101 mono-immunization groups showed severe weight loss, leading to euthanasia on day seven. In the SCVC101+OMN combination group, 50% of the mice required euthanasia due to critical weight loss, while the remaining half began weight restoration after day six (Figure 5B).

Overall, combined immunization with SCVC101 and OMN significantly improved the survival rate in mice (Figure 5B) and resulted in the lowest viral titer levels in the lungs (Figure 5C). These findings suggest that supplementing SCVC101 with OMN enhances the cross-protection against H1N1 influenza virus challenges.

## 4. Discussion

The substantial genetic and antigenic variability of influenza viruses necessitates continuous global surveillance to guide annual vaccine updates. Over the past decade, the efficacy of seasonal influenza vaccines has ranged between 10 and 60%, primarily determined by the degree of antigenic match between vaccine strains and circulating viral variants [29]. Thus, there is a high demand for better vaccines with broad cross-reactivity against influenza viruses. The highly conserved NP and M2e have the potential to induce cross-protection against influenza A viruses and are promising candidates for the development of broad-spectrum influenza vaccines [30,31,32,33]. In this study, we designed a heptameric nanoparticle vaccine based on conserved epitopes of the influenza virus. Our results indicate that the co-immunization with SCVC101 and OMN induced a stronger cellular immune response than SCVC101.

In this study, we concatenated six M2e peptide fragments (A/Cambodia/e0826360/2020) and replaced the two endogenous cysteines in each peptide with serine to prevent the spontaneous formation of structurally abnormal disulfide bonds [34]. Additionally, we employed immunoinformatics methods to predict all CTL and Th epitopes within the NP protein. To minimize the steric hindrance, we selected the peptide segments with the highest density of CTL and Th epitopes and combined them with the six repeatedly arranged M2e series connections. Ultimately, we chose the OVX313 heptamer as the vaccine carrier to construct OMN. Notably, the influenza vaccine OVX836, which utilizes the OVX313 heptamer as a carrier, has already progressed to Phase II clinical trials [22].

It is important to note that the combined immunization of SCVC101 and OMN was more effective in inducing heterologous cross-protection than SCVC101 alone. The mechanism behind this increased effectiveness likely involves antigen-epitope-mediated humoral and cellular immunity. In our experiments, OMN induced binding antibodies specific to multiple M2e subtypes following three immunizations in mice, suggesting its potential to provide cross-subtype protection. Although limited by experimental constraints, we did not evaluate the cellular immune responses. However, our findings showed that the NP2-1 peptide in the OMN vaccine group induced higher IFN-γ production in splenocytes compared to the M2e peptide. This phenomenon may be mechanistically attributed to IFN-γ’s critical role in Th1-type cellular immune responses, which promote CD8+ T-cell differentiation into CTLs. Previous studies have demonstrated that NP antigens can induce robust Th1-type immune responses in the lungs and significantly enhance the production of cytokines such as IFN-γ [19]. Additionally, prime-boost immunization with SCVC101 and OMN vaccines increased IFN-γ expression levels, suggesting that OMN may potentiate cellular immune responses of seasonal recombinant hemagglutinin influenza vaccines. Notably, combined immunization also significantly augmented the cellular immune responses against the M2e peptide, as evidenced by elevated IFN-γ and IL-4 levels. This is likely due to the inclusion of CD-A adjuvant in SCVC101, which, as a water-in-oil adjuvant containing squalene similar to MF59, can enhance both Th2 and Th1 immune responses while promoting antibody production [35]. IL-4 primarily mediates Th2-type humoral immune responses [36]. Furthermore, when mice were challenged with lethal doses of A/Victoria/2570/2019 (H1N1) and A/PuertoRico/8/34 (H1N1) viruses, combined immunization provided enhanced protective efficacy. Specifically, due to high antigenic matching, SCVC101 vaccination alone or in combination achieved 100% protection against A/Victoria/2570/2019. In contrast, SCVC101 monovaccination failed to protect against the mismatched A/PuertoRico/8/34 (H1N1) strain. Significantly, prime-boost immunization improved the protection against this mismatched virus, achieving a 50% survival rate while reducing viral loads in the lungs compared to SCVC101 alone. These results indicate that the predicted T-cell epitopes elicited cellular immune responses in mice, potentially conferring cross-protection against divergent influenza viruses. The combination of the SCVC101 and OMN vaccines induced more comprehensive serum antibodies and stronger cellular immunity, thereby providing cross-protection against heterologous H1N1 influenza viruses.

## 5. Conclusions

In this study, we demonstrated that OMN can serve as an effective complement to SCVC101, significantly enhancing its efficacy against H1N1 subtype influenza viruses. This strategy offers a safer transition in scenarios where there is an antigenic mismatch between vaccine strains and circulating epidemic strains, thereby bridging the gap and maintaining protection during such shifts. Moreover, our immune assessment method aligns with existing regulatory standards designed to evaluate HA-based influenza vaccines. This alignment not only supports the traditional serological criteria but also provides valuable insights into the efficacy evaluations of influenza vaccines that focus on cellular immunity. By integrating both humoral and cellular immune responses, our findings could contribute to a more comprehensive understanding of influenza vaccine efficacy and potentially inform future vaccine development strategies.

However, this study also has several limitations, including limited evaluation of epitope-specific cellular responses and suboptimal protective efficacy against H1N1/PR8-like viruses. To address these, future research will focus on further screening of optimal T-cell epitopes, complementing cellular immune assessments, and evaluating combinations of molecular adjuvants [37,38,39] to enhance the broader protective efficacy of the vaccine.

## Figures and Tables

**Table 1:** Predicted CTL epitopes of full-length NP proteins.

NetCTL1.2			IEDB-MHC I			SYFPEITHI		
Start Site	CTL Epitope Sequences	Score	Start Site	CTL Epitope Sequences	Score	Start Site	CTL Epitope Sequences	Score
**258**	**FLARSALIL**	**1.19**	**158**	**GMDPRMCSL**	**0.54**	185	GIGTMVMEL	25
135	HMMIWHSNL	1.13	307	LQNSQIYSL	0.52	**258**	**FLARSALIL**	**24**
**158**	**GMDPRMCSL**	**1.07**	**55**	**RLIQNSLTI**	**0.48**	**55**	**RLIQNSLTI**	**23**
**373**	**NMGSSTLEL**	**1.07**	**258**	**FLARSALIL**	**0.36**	**158**	**GMDPRMCSL**	**23**
**55**	**RLIQNSLTI**	**0.98**	**48**	**KLSDHEGRL**	**0.36**	336	AAFEDLRLL	23
307	LQNSQIYSL	0.95	**189**	**MVMELIRMI**	**0.30**	**373**	**NMGSSTLEL**	**23**
**189**	**MVMELIRMI**	**0.94**	225	ILKGKFQTA	0.25	**48**	**KLSDHEGRL**	**22**
**357**	**KLSTRGVQI**	**0.84**	**373**	**NMGSSTLEL**	**0.23**	256	LIFLARSAL	22
328	LVWMACHSA	0.78	31	KMIDGIGRF	0.19	**342**	**RLLSFIRGT**	**22**
**48**	**KLSDHEGRL**	**0.76**	**357**	**KLSTRGVQI**	**0.19**	60	SLTIEKMVL	21
185	GIGTMVMEL	0.72	185	GIGTMVMEL	0.17	262	SALILRGSV	21
**342**	**RLLSFIRGT**	**0.68**	**342**	**RLLSFIRGT**	**0.13**	**357**	**KLSTRGVQI**	**21**
41	IQMCTELKL	0.67	336	AAFEDLRLL	0.10	146	ATYQRTRAL	20
188	TMVMELIRM	0.62	135	HMMIWHSNL	0.08	**189**	**MVMELIRMI**	**20**

The bold sequences respresent the epitopes predicted by all the four immune informatics platforms used in this study.

**Table 2:** T_h_ epitope prediction from full-length NP protein.

IEDB-MHCII			RANKPEP		
Start Site	Th Epitope Sequences	Rank	Start Site	Th Epitope Sequences	Score
254	EDLIFLARSALILRGS	0.44	385	YWAIRTRSG	19.60
253	IEDLIFLARSALILRG	0.50	97	YRRVDGKWM	16.63
255	DLIFLARSALILRGSV	0.59	10	YEQMETDGD	15.89
252	EIEDLIFLARSALILR	0.79	423	STIMAAFTG	15.64
256	LIFLARSALILRGSVA	0.94	164	CSLMQGSTL	15.52
304	FKLLQNSQIYSLIRPN	1.10	304	FKLLQNSQI	13.78
300	GIDPFKLLQNSQIYSL	1.10	207	WRGENGRKT	12.25
301	IDPFKLLQNSQIYSLI	1.10	25	IRASVGKMI	12.14
251	AEIEDLIFLARSALIL	1.20	258	FLARSALIL	11.75
302	DPFKLLQNSQIYSLIR	1.20	273	KSCLPACAY	11.62
303	PFKLLQNSQIYSLIRP	1.20	120	WRQANNGED	11.33
298	LVGIDPFKLLQNSQIY	1.40	219	YDRMCNILK	11.26
297	SLVGIDPFKLLQNSQI	1.40	148	YQRTRALVR	10.79
299	VGIDPFKLLQNSQIYS	1.40	445	IRMMEGAKP	10.45
257	IFLARSALILRGSVAH	1.70	341	LRLLSFIRG	10.05

**Table 3:** M2e amino acid sequences of different influenza A virus strains.

Influenza Strains	Subtype	M2e Amino Acid Sequence
M2e in OMN nanoparticles(A/Cambodia/e0826360/2020 (H3N2))	H3N2	**SLLTEVETPIRNEWGSRSNDSSD**
A/Puerto Rico/30/2022	H1N1	**SLLTEVETP** T **R** S **EW** EC **R** CSG **S** N **D**
A/Cambodia/e0826360/2020	H3N2	**SLLTEVETPIRNEWG** C **R** C **NDSSD**
A/Guangdong-Shenzhen/1/2011	H5N1	**SLLTEVETP** T **RNEW** EC **R** CS **DSSD**
A/Jiangsu/602/2021	H9N2	**SLLTEVETP** T **R** TG **W** ECNCSG **SSD**
A/Jiangxi-Donghu/346-2/2013	H10N8	**SLLTEVET** LTKTG **W** ECNCSG **SSD**

The M2e amino acid sequences of different influenza A viruses (H1N1, H3N2, H5N1, H9N2, H10N8) were compared and aligned with our antigen sequences, with the diversified sequences underlined while the conserved sequences bolded.

**Table 4:** Amino acid sequences of NP peptides of different influenza A virus strains.

Influenza Strains	Subtype	NP Amino Acid Sequence
A/Puerto Rico/30/2022	H1N1	NP2-1 (**AEIEDLIFLARSALILRGSVAHKS**)
		NP2-2 (**SLVGIDPFKLLQNSQ**VV**SL**M**RPNEN**)
		NP2-3 (**R**VS**SFIRG**K**KV**I**PRGKLSTRGVQIA**)
		NP2-4 (T**M**D**S**N**TLELRS**R**YWAIRTRSGGNTN**)
NP in OMN nanoparticles	H3N2	NP2-1 (**AEIEDLIFLARSALILRGSVAHKS**)
		NP2-2 (**SLVGIDPFKLLQNSQIYSLIRPNEN**)
		NP2-3 (**RLLSFIRGTKVSPRGKLSTRGVQIA**)
		NP2-4 (**NMGSSTLELRSGYWAIRTRSGGNTN**)
A/Guangdong-Shenzhen/1/2011	H5N1	NP2-1 (**AEIEDLIFLARSALILRGSVAHKS**)
		NP2-2 (**SLVGIDPFRLLQNSQ**VF**SLIRPNEN**)
		NP2-3 (**R**VS**SFIRGT**R**V**V**PRG**Q**LSTRGVQIA**)
		NP2-4 (T**M**D**S**N**TLELRS**R**YWAIRTRSGGNTN**)
A/Jiangsu/602/2021	H9N2	NP2-1 (**AEIEDLIFLARSALILRGSVAHKS**)
		NP2-2 (**SLVGIDPF**R**LLQNSQ**VF**SLIRPNEN**)
		NP2-3 (**R**VS**SFIRGT**RMV**PRG****Q****LSTRGVQIA**)
		NP2-4 (T**M**D**S**N**TLELRS**R**YWAIRTRSGGNTN**)
A/Jiangxi-Donghu/346-2/2013	H10N8	NP2-1 (**AEIEDLIFLARSALILRGSVAHKS**)
		NP2-2 (**SLVGIDPF**R**LLQNSQ**VF**SLIRPNEN**)
		NP2-3 (**R**VS**SFIRGT**RMV**PRG**Q**LSTRGVQIA**)
		NP2-4 (A**M**D**S**N**TLELRS**R**YWAIRTRSGGNTN**)

The NP amino acid sequences of different influenza A viruses (H1N1, H3N2, H5N1, H9N2, H10N8) were compared and aligned with our antigen sequences, with the diversified sequences underlined while the conserved sequences bolded.

## Data Availability

The raw data supporting the conclusions of this article will be made available by the authors on request.
